# Concordance between TP53 alterations in blood and tissue: impact of time interval, biopsy site, cancer type and circulating tumor DNA burden

**DOI:** 10.1002/1878-0261.12672

**Published:** 2020-04-07

**Authors:** Cheyennedra C. Bieg‐Bourne, Ryosuke Okamura, Razelle Kurzrock

**Affiliations:** ^1^ Center for Personalized Cancer Therapy Moores Cancer Center University of California San Diego La Jolla CA USA

**Keywords:** cancer, concordance, ctDNA, genomics, TP53

## Abstract

We examined the impact of spatial, temporal, histologic, and quantitative factors on concordance between *TP53* alterations in tissue DNA vs in circulating tumor DNA (ctDNA). Four hundred and thirty‐three patients underwent next‐generation sequencing (NGS) in which both tissue and blood samples were evaluated. *TP53* was detected in 258 of 433 patients (59.6%); 215 had tissue *TP53* alterations (49.7%); 159, ctDNA (36.7%); and 116, both tissue and ctDNA (27.8%). Overall concordance rate between ctDNA and tissue biopsies for *TP53* alterations was 67.2%; positive concordance was 45.0%. Overall concordance for *TP53* did not vary among patients with ≤ 2 months vs > 6 months between test samples; however, positive concordance trended higher when time intervals between test samples were shorter, suggesting that the lack of difference in overall concordance may be due to the large number of negative/negative tests. There was a trend toward higher overall concordance based on biopsy site (metastatic vs primary) (*P* = 0.07) and significantly higher positive concordance if the tissue biopsy site was a metastatic lesion (*P* = 0.03). Positive concordance significantly decreased in noncolorectal cancer patients vs colorectal cancer patients (*P* = 0.02). Finally, higher %ctDNA was associated with higher concordance rates between blood and tissue (*P* < 0.001). Taken together, these data indicate that both blood and tissue DNA sequencing are necessary to evaluate the full scope of *TP53* alterations, and that concordance rates may be related to multiple factors including, but not limited to, amount of ctDNA, histologic context, and site of tissue biopsy.

## Graphical abstract

This study presents evidence that the evaluation of the full spectrum of *TP53 *alterations in patients with diverse malignancies requires sequencing of both blood‐derived circulating tumor DNA and tissue DNA. Multiple factors can influence positive concordance rates between the tests, including site of tissue biopsy, %ctDNA, time between tissue biopsy and blood sample, and histologic context.

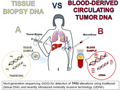

**Abbreviations**
ctDNA: Circulating tumor DNA
GI: Gastrointestinal
MAF: Mutant allele frequency
NGS: Next‐Generation Sequencing
UCSD: University of California San Diego
VUS: Variants of unknown significance

## 1 Introduction

Cancer genome sequencing is enabling the use of precision medicine in clinical oncology. Numerous genetic aberrations drive tumor progression and characteristics (MacConaill, 2013; Schwaederlé *et al.*, 2016). The detection of actionable alterations through genomic sequencing facilitates the development of cancer treatment and understanding the underlying biology of the neoplasm. Traditional genomic profiling of cancer utilizes tissue biopsies, and several studies continue to utilize these conventional methods to determine the actionability of alterations (Agarwal *et al.*, 2018; Goodman *et al.*, 2017; Thierry *et al.*, 2017). However, tumor evolution through time and space poses a challenge to developing a more complete portrait of the tumor (Swanton, 2012).

Innovative noninvasive technologies deploying liquid biopsy to analyze blood‐derived circulating tumor DNA (ctDNA) have recently been exploited in the clinical setting (Han *et al.*, 2017; MacConaill, 2013; Schwaederlé *et al.*, 2017; Wyatt *et al.*, 2017). For example, Schwaederlé et al revealed that the majority of patients (61.5%) with lung cancer who underwent ctDNA analysis had at least one potentially targetable alteration (Schwaederlé *et al.*, 2017). Overall concordance rates for tissue and ctDNA varied between 70 and 93 percent, depending on the alteration (Schwaederlé *et al.*, 2016; Schwaederlé *et al.*, 2017).

*TP53* alterations are ubiquitous in cancer and are detected in about 40% of malignancies (Solomon, *et al.*, 2018; Soussi *et al.*, 2006). Being the most frequently mutated gene in cancers, investigators have closely examined *TP53* to determine its relationship with outcomes (Kadia *et al.*, 2016; Robles and Harris, 2010; Said *et al.*, 2013; Said *et al.*, 2014; Schwaederlé *et al.*, 2017; Solomon *et al.*, 2018; Soussi, *et al.*, 2006; Sun *et al.*, 2018; Villaflor *et al.*, 2016). Research suggests that *TP53* mutations may be an indicator of a poor prognosis (Poeta *et al.*, 2007).

With the recent increase in clinical oncology research moving to more minimal invasive and efficient technologies, blood‐derived ctDNA testing has become more appealing. Studies have revealed that next‐generation sequencing (NGS) frequently detects *TP53* alterations in both tissue and ctDNA (Robles and Harris, 2010; Said *et al.*, 2013, 2014; Solomon *et al.*, 2018; Soussi, *et al.*, 2006; Sun *et al.*, 2018). Herein, we examine the impact of temporal separation on concordance between *TP53* mutations in blood vs tissue, as well as associations between concordance and biopsy site, histology, and %ctDNA in 433 patients with diverse cancer types who underwent NGS of tissue and plasma‐derived ctDNA.

## 2 Materials and methods

### 2.1 Patients

Molecular profiles from tissue and liquid biopsy from 433 consecutive patients seen at the University of California San Diego (UCSD), Moores Cancer Center (La Jolla, CA, USA), were evaluated. Demographic characteristics of each patient such as age, gender, and cancer diagnosis were obtained. The current analysis was performed on those eligible patients who had both tissue and liquid biopsies interrogated starting in June 2014. This study was performed, and consents obtained in accordance with UCSD Institutional Review Board guidelines (NCT02478931). The experiments were undertaken with the understanding and written consent of each subject. The study methodologies conformed to the standards set by the Declaration of Helsinki, and the study methodologies were approved by the local ethics committee.

### 2.2 Molecular profile by next‐generation sequencing (NGS)

Profiling of both tissue and ctDNA was performed in a clinical laboratory improvement amendment‐certified laboratory. Variants of unknown significance (VUS) were excluded from the analysis.

#### 2.2.1 Tissue

Next‐generation sequencing was performed by Foundation Medicine (Cambridge, MA, USA) (236–315 genes) as previously described (Frampton *et al.*, 2013). Libraries were sequenced to high, uniform median coverage (> 500 times). Tumor DNA was evaluated for genomic anomalies including multiple alterations per gene, deletions, amplifications, short variants, insertions, base substitutions, copy number alterations, and fusions/rearrangements. All samples had a minimum of 20% tumor cells. Optimization of mutation detection was exemplified by testing base substitutions, insertions, deletions, rearrangements, and amplifications at ≥ 5% mutant allele frequency (MAF) and indels with a ≥ 10% MAF with ≥ 99% accuracy.

#### 2.2.2 ctDNA

Digital sequencing (54–73 genes) was performed by Guardant Health, Inc. (Redwood City, CA, USA; Guardant360, http://www.guardanthealth.com/guardant360/) (Lanman *et al.*, 2015). VUSs were excluded in this analysis.

Circulating tumor DNA was extracted from blood collected in 10‐mL Streck tubes, and 5–30 ng of ctDNA was prepared for sequencing. All ctDNA was sequenced, including the germline and the somatic ctDNA. All sequence‐based mutations were assessed for allele frequency. Allele frequencies were generally at about 100% (homozygous single‐nucleotide polymorphism), about 50% (heterozygous germline), and < 5% (somatic fraction). In addition to the allele frequency, the specific alteration was also evaluated using the Database of Short Genetic Variation and COSMIC database to distinguish germline from somatic mutation. The fractional concentration for a somatic alteration is calculated as the fraction of ctDNA harboring that mutation in a background of wild‐type ctDNA fragments at the same nucleotide position. The analytic sensitivity reaches the detection of one to two single‐mutant fragments from a 10 mL blood sample (0.1% limit of detection), and analytic specificity is over 99.9999%.

### 2.3 Alteration concordance

Concordance among alterations identified through tissue biopsy samples and alterations identified through ctDNA liquid or blood‐derived biopsy samples were examined at the gene level unless otherwise specified, in which case it was examined at the locus level. If there was more than one time point of tissue or ctDNA NGS, the time points closest together for each patient were chosen. Overall concordance rates and Kappa values were performed to determine concordance between tissue and ctDNA. The Kappa agreement categories, 1 (perfect agreement) to 0 (no agreement), were used for interpretation of the analysis.

### 2.4 Statistical analysis

Patient descriptive characteristics including cancer histology, gender, age, time at diagnosis, date of sample collection, and age at metastatic disease were analyzed and summarized. Statistical differences in overall concordance between two groups were determined by performing Fisher’s exact test. All statistical analyses were performed using r Studio version 1.1.383 (R Studio, Inc., Boston, MA, USA) and sas software version 9.4 (SAS Inc., Cary, NC, USA).

## 3 Results

### 3.1 Patient demographics

The current study reviewed and analyzed the genomic profiles of 433 patients with diverse cancer types seen at UCSD Moores Cancer Center who had both tissue and liquid biopsies analyzed (Table 1). The median age at the time of ctDNA liquid biopsy was 62 years (range, 19–93). The majority of participants were women (237, 54.7% of patients). The analysis revealed that the most common diagnoses were lung cancer (78, 18.0%), brain cancer (56, 12.9%), gastrointestinal (GI), colorectal (54, 12.5%), others/unknown primary (52, 12.0%), GI, noncolorectal (50, 11.5%), and breast cancer (50, 11.5%). Fifty‐four of 433 patients (12.5%) had colorectal cancer at the time of interrogation. Of 433 patients who underwent tissue and liquid biopsies, 385 (88.9%) had metastatic disease at the time of ctDNA test and 328 (75.8%) had metastatic disease at the time of tissue biopsy.

**Table 1:** Patient demographics among (*N* = 433) patients who underwent tissue and blood‐derived ctDNA NGS^a^.

Characteristic	Number of patients
Gender (*N*, %)	
Women	237 (54.7%)
Men	196 (45.3%)
Median age at time of ctDNA (Range)	62 years (19–93)
Diagnosis (*N*, %)	
Lung cancer	78 (18.0%)
Brain cancer	56 (12.9%)
GI, Colorectal	54 (12.5%)
GI, Noncolorectal	50 (11.5%)
Breast cancer	50 (11.5%)
Hepato‐pancreato‐biliary	40 (9.2%)
Head and neck cancer	31 (7.2%)
Gynecologic cancer	22 (5.1%)
Others/Unknown primary	52 (12.0%)
Time interval between blood draw and tissue biopsy, months	
≤ 2	165 (38.1%)
> 2–6	69 (15.9%)
> 6	199 (46.0%)
Disease stage at time of blood draw (*N*, %)	
Metastatic or locally advanced	385 (88.9%)
Not metastatic	48 (11.1%)
Disease state at time of tissue biopsy (*N*, %)	
Metastatic or locally advanced	328 (75.8%)
Not metastatic	105 (24.2%)

^a^ Data derived from database described by Mardinian *et al. *(2019).

### 3.2 Common genomic alterations in tissue and ctDNA

**Fig. 1:**
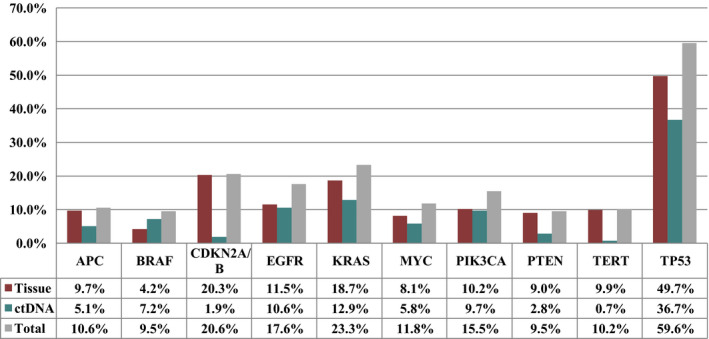
The 10 most common characterized alterations (VUS excluded) among tissue biopsy and blood‐derived ctDNA (*N* = 433 total patients). If there were two alterations in one gene in a patient, only one was counted.

Figure 1 depicts the most common characterized alterations among tissue‐derived DNA and blood‐derived ctDNA in the 433 total patients.

**Fig. 2:**
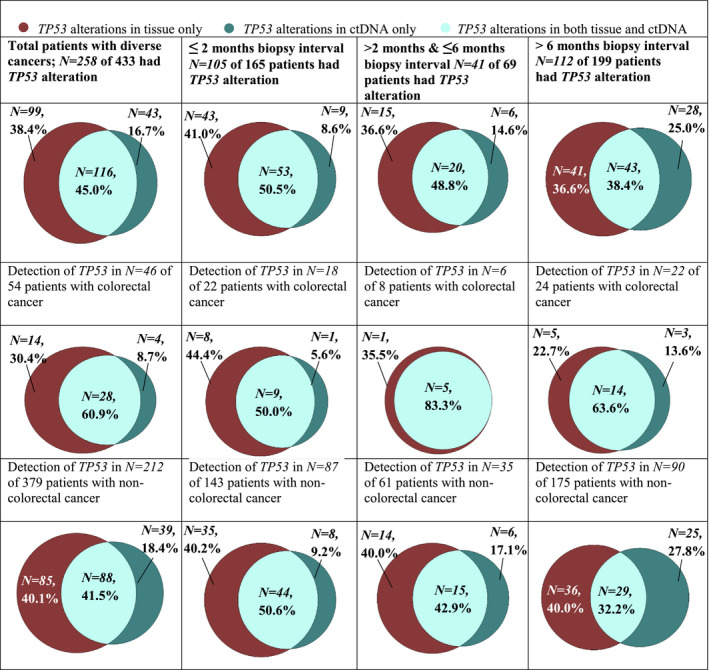
Alteration detection performed by tissue and ctDNA tests. Venn diagrams represent the proportion of patients who had *TP53* detected in only tissue, in both tissue and ctDNA, and only in ctDNA. Concordance was examined at the gene level. If there was more than one time point of tissue or ctDNA NGS, the time points closest together for each patient were chosen.

Altogether, 59.6% (*N* = 258/433) had at least one *TP53* alteration detected (in blood, tissue or both). As displayed in Fig. 2 and Table 2, a total of 116 patients (26.8% of 433) had *TP53* alterations detected in both ctDNA and tissue; 99 (22.9% of 433 patients) and 43 (9.9% of 433) patients had *TP53* detected in tissue only and ctDNA only, respectively.

**Table 2:** Overall and positive concordance of *TP53*. Concordance of *TP53* alterations (*N* = 433 patients) stratified by time between tissue biopsy and blood draw (≤ 2, 2–6, and > 6 months between tissue biopsy and blood draw) as well as overall concordance; temporal and spatial effects and positive concordance among patients with (≤ 2 months, 2–6 months, and > 6 months between tissue biopsy and blood‐derived ctDNA as well as primary vs metastatic site for tissue biopsy comparison.

All patients (*N* = 433)					
	Tissue DNA results		Overall concordance rate^a^	Kappa (SE)	Positive concordance rate^b^
	Positive	Negative			
ctDNA results					
Positive	116	43	67.2%	0.34 (0.04)	45.0%
Negative	99	175			

Temporal and spatial effects on concordance								
	Test results (ctDNA/ tissue DNA)			Overall concordance^a^			Positive concordance^b^	
	(+/+)	(+/− plus −/+)	(−/−)	Rate	Kappa (SE)	*P‐*value	Rate	*P‐*value
Time interval between blood draw and tissue biopsy								
≤ 2 months (*N* = 165)	53	52	60	68.5%	0.39 (0.06)	0.58 (≤ 2 vs > 6) 0.47 (≤ 6 vs > 6)	50.5%	0.08 (≤ 2 vs > 6) 0.08 (≤ 6 vs > 6)
> 2–6 months (*N* = 69)	20	21	28	69.6%	0.39 (0.11)		48.8%	
> 6 months (*N* = 199)	43	69	87	65.3%	0.27 (0.07)		38.4%	
Tissue biopsy site								
Primary (*N* = 228)	53	84	91	63.2%	0.27 (0.06)	0.07	38.7%	0.03
Metastatic (*N* = 205)	63	58	84	71.7%	0.43 (0.06)		52.1%	

a Overall concordance = (++) + (−−)/ Total. ^b^ $\text{Positive}\;\text{concordance}=\frac{\left(\text{positive in both ctDNA and tissue DNA}\right)}{\left(\text{positive in either ctDNA or tissue DNA or in both}\right)}.$

When examining alterations in ctDNA or tissue DNA or both, *KRAS* alterations were detected in 23.3% of 433 patients (Fig. 1); *CDKN2A/B*, in 20.6% of patients; *EGFR*, in 17.6% of patients; and *PIK3CA*, in 15.5% of 433 patients. The most common alterations discerned in tissue biopsies include *TP53* (49.7%), *CDKN2A* (20.3%), *KRAS* (18.7%), and *EGFR* (11.5%); the most common alterations detected in blood‐derived ctDNA were *TP53* (36.7%), *KRAS* (12.9%), *EGFR* (10.6%), *PIK3CA* (9.7%), and *BRAF* (7.2%).

### 3.3 Overall and positive concordance between blood‐derived ctDNA testing and tissue NGS

Overall *TP53* alteration concordance was seen in 67.2% of patients (includes 116 patients that were positive for *TP53* in both tissue and blood as well as 175 patients who were negative for *TP53* in both tissue and blood) (Table 2 and Fig. 2).

**Fig. 3:**
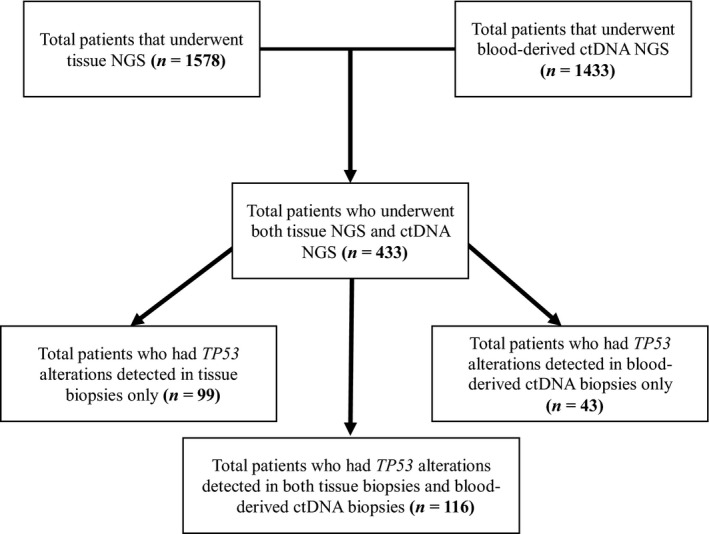
Consort diagram that displays the detection of *TP53* alteration in both tissue and blood‐derived ctDNA NGS.

Of the 258 patients with *TP53* mutations, 116 (45.0%) had *TP53* alterations detected in both tissue biopsies and blood‐derived cDNA biopsies (positive concordance). Moreover, 99/258 patients had *TP53* alterations detected in tissue biopsies only, and 43 had *TP53* alterations detected in blood‐derived ctDNA only (Table 2, Figs 2 and 3).

### 3.4 Temporal effects: Concordance between ctDNA and tissue DNA was not different for patients who had sampling over 6 months apart versus less than 2 months apart

For 165 patients in whom the tissue vs ctDNA was obtained ≤ 2 months apart, the concordance rate was 68.5%; for the 199 patients who had tissue vs ctDNA samples more than 6 months apart, 130 (65.3%) were concordant (*P* = 0.47) (Table 2). The lack of difference in concordance may have been due to the high number of negative samples. Indeed, there was a trend for higher positive concordance when the time interval between blood and tissue sampling was shorter (*P* = 0.08 and *P* = 0.08, depending on time intervals used) (Table 2).

### 3.5 Spatial effects: Concordance rates between ctDNA and tissue trended higher if tissue biopsies were taken from metastatic versus primary sites

As displayed in Table 2, there was a trend toward higher overall concordance if the tissue biopsy was from metastatic disease vs from the primary (71.7%, vs 63.2%, *P* = 0.07). A significant difference was further observed in positive concordance between metastatic vs primary disease tissue biopsy site (52.1% vs 38.7%, *P* = 0.03).

### 3.6 Colorectal (versus other) cancer had higher positive concordance between ctDNA and tissue

Overall concordance did not differ when stratifying patients as colorectal cancer (*N* = 54) vs other diagnoses (*N* = 379, Table S1). However, positive concordance rate was higher for *TP53* alterations in colorectal cancer patients vs other malignancies (60.9% vs 41.5%; *P* = 0.02).

### 3.7 Higher %ctDNA correlated with better overall concordance

After dichotomizing by %ctDNA using the median %ctDNA for *TP53* alterations (1.50%), concordance rates significantly differed between < 1.50% ctDNA and ≥ 1.50% ctDNA, 59.5% and 86.3%, respectively (*P* < 0.001, Table S2). When only patients with ≤ 6 months were included, this significant difference was retained (*P* = 0.01, Table S3).

### 3.8 Accuracy of ctDNA for tissue DNA among TP53 alterations

Sensitivity of ctDNA for tissue DNA *TP53 r*esults was 54%; specificity was 80.3%. Positive predictive value was 73%, and negative predictive value was 63.9% (Table S4). Longer time intervals between blood and tissue sampling reduced the positive predictive value (*P* = 0.002) and showed a trend toward reduction of the specificity (*P* = 0.09).

Sensitivity of tissue DNA for ctDNA was 73%; specificity was 63.9%. Positive predictive value was 54%, while negative predictive value was 80.3% (Table S5). Longer time intervals between blood and tissue sampling reduced the positive sensitivity (*P* = 0.002) and showed a trend toward reduction of the negative predictive value (*P* = 0.09).

### 3.9 TP53 loci alterations

The most common *TP53* molecular alterations examined were *TP53* R248W (10/258), *TP53* R248Q (9/258), *TP53* R282W (9/258), *TP53* G245S (8/258), and *TP53* R175H (7/258); overall concordance between tissue and blood ctDNA was 97.3%, 98.1%, 97.7%, 98.1%, and 98.4%, respectively (data not shown). However, positive concordance was much lower between 30% and 44.4% (Fig. S1), keeping in mind that number of patients was small for each type of *TP53* alteration.

## 4 Discussion

Blood‐derived ctDNA and tissue DNA are frequently analyzed by NGS for determining diagnosis, prognosis, and treatment. Previous studies have described the frequency of *TP53* alteration detection using tissue DNA and blood‐derived NGS separately (Robles and Harris, 2010; Said *et al.*, 2013, 2014; Solomon *et al.*, 2018; Soussi, *et al.*, 2006; Sun *et al.*, 2018). However, there is a paucity of research determining concordance between *TP53* alterations in blood‐derived ctDNA vs tissue DNA. The current study evaluates temporal (determined by time interval between tissue DNA and blood‐derived ctDNA), cancer histopathology‐related (examining colorectal vs noncolorectal cancer), spatial (determined by tissue extracted from primary vs metastatic site), and quantitative (reflected by %ctDNA) effects on *TP53* alteration concordance among 433 patients who underwent blood‐derived ctDNA and tissue biopsy tests.

Our analysis revealed that the most common alterations detected in both tissue biopsy and blood‐derived ctDNA were in the *TP53* gene (49.7% and 36.7%, respectively); altogether, 59.6% of patients had ≥ 1 *TP53* alteration in blood, tissue, or both. These frequencies are consistent with previous studies in the literature (Kadia *et al.*, 2016; Robles and Harris, 2010; Said *et al.*, 2013, 2014; Schwaederlé *et al.*, 2017; Solomon *et al.*, 2018; Soussi, *et al.*, 2006; Sun *et al.*, 2018; Villaflor *et al.*, 2016). Other common alterations in ctDNA, tissue, or both were in the *KRAS* (23.3%), *CDK2A/B* (20.6%), *EGFR* (17.6%), and *PIK3CA* genes (15.5%) (Fig. 1). For the most part, alteration frequencies were similar (but not identical) in ctDNA and tissue, with a few notable exceptions: *CDKN2A/B* (20.3% for tissue and 1.9% for ctDNA), *PTEN* (9.0% for tissue and 2.8% for ctDNA), and *TERT* (9.9% for tissue and 0.7% for ctDNA). For *CDKN2A/B,* the discrepancy is most likely due to the inability to discern allelic loss in earlier ctDNA sequencing panels. Other reasons for discrepant result could be technological issues, sensitivity of tissue vs ctDNA testing, and suppression of ctDNA alterations by treatment.

This study revealed that the overall concordance rate for *TP53* in tissue and ctDNA was 67.2% and the positive concordance rate was 45.0%; the latter suggests that some of the overall concordance rate was driven by the samples that were negative in both tissue and blood.

Overall concordance did not change with time interval between blood draw and tissue biopsy (*P* = 0.58, ≤2 vs > 6 months apart, Table 2). Similar to the current study, overall concordance did not vary with time interval between blood and tissue biopsy (*P* = 0.67) for patients who had *KRAS* alterations (Mardinian *et al.*, 2019). However, these observations differ from those previously reported wherein the median time interval between tissue biopsy and blood draw was 2.7 vs 14.4 months (*P* = 0.006) for patients who had ≥ 1 alteration (vs no alterations) in common between blood and tissue albeit this study looked at multiple alterations and not just *TP53* (Schwaederlé *et al.*, 2016). The reason that the time interval between testing did not affect the concordance rate for *TP53* is unclear, but could be due to stability of the *TP53* alteration among metastatic sites.

In assessing spatial effects, there was a trend toward greater *TP53* alteration concordance when the tissue biopsy was from a metastatic vs a primary site (*P* = 0.07); this was especially pronounced with positive concordance (52.1% vs 38.7%) (*P* = 0.03, Table 2). We previously examined the impact of primary vs metastatic site for ctDNA vs tissue concordance for *KRAS* mutations (Mardinian *et al.*, 2019) and did not find a difference, but the much smaller number of patients with *KRAS* mutations might have limited the ability to find differences. Similar to our observations, a previous study assessing heterogeneity in ctDNA genomic portfolios in gastroesophageal cancers also reported that several genomic alterations were 88% concordant between metastatic tissue and ctDNA even when primary tumor and metastatic sites had discordant results (Pectasides *et al.*, 2018). Taken together, these data suggest that disease heterogeneity creates important differences in results depending on sampling and that interrogation of metastatic lesions and/or ctDNA for therapeutic decision making warrants additional evaluation.

Regarding histologic effects, overall *TP53* concordance of blood vs tissue did not differ between colorectal and noncolorectal cancer, but positive concordance was higher in colorectal cancer (60.9% vs 41.5%; *P* = 0.02). Previous studies have also shown high concordance for blood and tissue alterations, such as those in the *KRAS, NRAS*, *APC,* and *BRAF* genes, in colorectal cancer (Grasselli *et al.*, 2017; Gregg *et al.*, 2018; Kato *et al.*, 2019; Mardinian *et al.*, 2019).

Our study revealed that overall concordance was also significantly higher when the %ctDNA was higher (Tables S2 and S3). When only patients with ≤ 6 months between tests were included, this phenomenon remained significant. It may be that the higher concordance is because higher %ctDNA reflects greater tumor burden or increases ease of detection of blood alterations.

Our analysis showed that the positive predictive value of ctDNA for tissue DNA was 73.0% for detecting *TP53*, revealing that, of the *TP53* alterations detected ctDNA tests, 73.0% were also positive by tissue. The positive predictive value of ctDNA for tissue was significantly reduced when ctDNA and tissue DNA samples were > 6 months apart vs ≤ 2 months apart (60.6% vs 85.5%, *P* = 0.002). The positive predictive value of tissue for ctDNA positivity was 54.0%, which shows that, of the *TP53* alterations detected tissue DNA tests, 54.0% were positive by ctDNA. There was no difference detected between ctDNA and tissue DNA when the samples were taken ≤ 2 months apart vs > 6 months apart (55.2% vs 51.2%, *P* = 0.65); therefore, greater time intervals between ctDNA and tissue DNA dates did not significantly affect the detection of *TP53*. Taken together, these observations suggest that, with time, new *TP53* mutations may emerge in ctDNA that were not in the original tissue DNA, perhaps coming from new metastatic sites or evolved clones; however, the original tissue *TP53* alterations remain detectable in the ctDNA, suggesting that they do not disappear with time. Even so, overall, the negative predictive value and specificity of ctDNA for tissue (equals specificity and negative predictive value of tissue DNA for ctDNA, respectively) remained consistent (63.9% and 80.3%) irrespective of the time interval between the tests (Tables S4 and S5).

Several limitations of the current study should be noted. For example, confounders for *TP53* alteration detection were not analyzed in this study. It is possible that the detection of *TP53* could be altered by therapeutic treatments (Kadia *et al.*, 2016; Robles and Harris, 2010; Said *et al.*, 2013, 2014; Wheler *et al.*, 2016). Further, the number of samples collected for each cancer type varied based on the physician ordering the test and, hence, while the diverse cancer types included herein may suggest generalizability of the observations, we were not able to analyze differences between histologies. A significant minority of our patients had brain tumors, and the low %ctDNA in these cancers might have led to a bias in examining blood/tissue concordance. Finally, the use of different platforms for NGS of blood and tissue may have confounded some of the results, as it is currently not clear the extent to which different platforms can be validated against each other, even when platforms are clinical grade. For instance, Stetson and colleagues (Stetson *et al.*, 2019) previously tested four ctDNA platforms and found a range of sensitivity (38–89%) and positive predictive value (36–80%), with most cross‐vendor discordance observed below 1% variant allele frequency.

In summary, *TP53* alterations were detected in 59.6% of patients with diverse cancer types (36.7% of patients were positive in the blood test, and 49.7% were positive in tissue). The overall concordance between ctDNA and tissue DNA was 67.2%. Our analysis revealed that time between tests did not significantly affect the concordance of *TP53* alterations. On the other hand, spatial effects produced a trend for higher overall concordance when the tissue biopsy was taken from metastatic vs primary sites. Finally, higher %ctDNA was associated with higher concordance rates between blood and tissue. Taken together, these observations suggest that both blood and tissue DNA sequencing are required to determine the full extent of *TP53* alterations, and that the concordance rates may be related to multiple factors such as amount of ctDNA and site of tissue biopsy.

## 5 Conclusions

The current study demonstrates that evaluating the full spectrum of TP53 alterations in patients with diverse malignancies requires sequencing of both blood‐derived ctDNA and tissue DNA, as also highlighted in the joint ASCO and CAP report (Merker *et al.*, 2018). Multiple factors can influence positive concordance rates between the tests, including site of tissue biopsy, %ctDNA, time between tissue biopsy and blood sample, and histologic context.

## Conflict of interest

RK has research funding from Incyte, Genentech, Merck Serono, Pfizer, Sequenom, Foundation Medicine, Guardant Health, Grifols, and Konica Minolta, and Omniseq, as well as consultant fees from LOXO, X‐Biotech, Actuate Therapeutics, Roche and NeoMed. She receives speaker fees from Roche, and has equity in IDbyDNA, and CureMatch, Inc.

## Author contributions

CCB and RK developed the study conception and design. CCB and RO performed data acquisition/statistical analysis. All authors were involved in data interpretation, drafting the manuscript or revising it critically, and final approval of the manuscript.

## Supporting information


**Table S1** **.** Concordance for *TP53* alterations in tissue versus ctDNA by type of cancer.
**Table S2** **.** Concordance between tissue and blood *TP53* alterations based on %ctDNA (dichotomized at median %ctDNA for *TP53* alterations).
**Table S3** **.** Concordance between tissue and blood *TP53* alterations based on %ctDNA when there was ≤ 6 months between tissue biopsy and blood sample (dichotomized at median %ctDNA for *TP53* alterations).
**Table S4** **.** Accuracy of ctDNA for tissue DNA results.
**Table S5** **.** Accuracy of tissue DNA for ctDNA results.
**Fig. S1** **.** Most common locus‐specific alterations detected among 258 patients with *TP53* detected.

## Data Availability

The data supporting the findings of this study are available upon request from the corresponding author. The data are not publicly available due to privacy or ethical restrictions.
