# Supplementary material for: Concordance between TP53 alterations in blood and tissue: impact of time interval, biopsy site, cancer type and circulating tumor DNA burden
Source: Mol Oncol. 2020 Apr 7;14(6):1242–51. doi: 10.1002/1878-0261.12672 (PMC7266274; doi:10.1002/1878-0261.12672)
Supplement: Supplementary file 1 — Table S1 . Concordance for TP53 alterations in tissue versus ctDNA by type of cancer. Table S2 . Concordance between tissue and blood TP53 alterations based on %ctDNA (dichotomized at median %ctDNA for TP53 alterations). Table S3 . Concordance between tissue and blood TP53 alterations based on %ctDNA when there was ≤ 6 months between tissue biopsy and blood sample (dichotomized at median %ctDNA for TP53 alterations). Table S4 . Accuracy of ctDNA for tissue DNA results. Table S5 . Accuracy of tissue DNA for ctDNA results. Fig. S1 . Most common locus‐specific alterations detected among 258 patients with TP53 detected. [file MOL2-14-1242-s001.docx]

**SUPPORTING INFORMATION**

**Supplemental Table 1:** Concordance for *TP53* alterations in tissue versus ctDNA by type of cancer.

| ***Type of cancer*** | ***(+/+)*** | ***(-/-)*** | ***(+/- plus -+)*** | ***Overall concordance rate*** | ***Kappa (SE)*** | ***P-value*** | ***Positive Concordance rate^a^*** | ***P-value*** |
| --- | --- | --- | --- | --- | --- | --- | --- | --- |
| **Colorectal cancer patients (N=54)** | 28 | 8 | 18 | 66.7% | 0.26 (0.13) | >0.99 | 60.9% | 0.02 |
| **Non-colorectal cancer patients (N=379)** | 88 | 167 | 124 | 67.3% | 0.33 (0.05) |  | 41.5% |  |

^a^Positive concordance = $\frac{(positive in both ctDNA and tissue DNA)}{(positive in either ctDNA or tissue DNA or in both)}$

**Supplemental Table 2:** Concordance between tissue and blood *TP53* alterations based on %ctDNA (dichotomized at median %ctDNA for *TP53* alterations).^a^

|  |  | **Tissue DNA results** | |  | |  |
| --- | --- | --- | --- | --- | --- | --- |
| **<1.50% ctDNA (N=79)** | | *Positive* | *Negative* | **Overall concordance** | **Kappa (SE)** | **P-value** |
| **ctDNA results** | *Positive* | 47 | 32 | 59.5% | - | <0.001 |
|  | *Negative* | 0 | 0 |  |  |  |
| **≥1.50% ctDNA (N=80)** | | *Positive* | *Negative* | **Overall concordance** | **Kappa (SE)** |  |
| **ctDNA results** | *Positive* | 69 | 11 | 86.3% | - |  |
|  | *Negative* | 0 | 0 |  |  |  |

^a^Among 159 patients with *TP53* alterations in ctDNA, all patients were available for %ctDNA. In these 159 patients, the median %ctDNA of *TP53* alterations was 1.50% (range, 0.1% - 75.0%); the *Kappa* values could not be calculated due to ‘ctDNA negative = 0’. Note that concordance may be biased in this analysis because, by definition, in this analysis, all patients had *TP53* alterations in ctDNA. Analysis was performed using Fisher’s exact test.

**Supplemental Table 3:** Concordance between tissue and blood *TP53* alterations based on %ctDNA when there was ≤6 months between tissue biopsy and blood sample (dichotomized at median %ctDNA for *TP53* alterations).^a^

|  |  | **Tissue DNA results** | |  | |  |
| --- | --- | --- | --- | --- | --- | --- |
| **<2.0 %ctDNA (N=43)** | | *Positive* | *Negative* | **Overall concordance** | **Kappa (SE)** | **P-value** |
| **ctDNA results** | *Positive* | 31 | 12 | 72.1% | - | 0.01 |
|  | *Negative* | 0 | 0 |  |  |  |
| **≥2.0 % ctDNA (N=45)** | | *Positive* | *Negative* | **Overall concordance** | **Kappa (SE)** |  |
| **ctDNA results** | *Positive* | 42 | 3 | 93.3% | - |  |
|  | *Negative* | 0 | 0 |  |  |  |

^a^Among 88 patients with *TP53* alterations in ctDNA and with ≤6 months between studies, all patients were available for %ctDNA. In these 88 patients, the median %ctDNA for *TP53* alterations was 2.0% (range, 0.1% - 75.0%); the *Kappa* values could not be calculated due to ‘ctDNA negative = 0’. Note that concordance may be biased in this analysis because, by definition, in this analysis, all patients had *TP53* alterations in ctDNA. Analysis was performed using Fisher’s exact test.

**Supplemental Table 4**: Accuracy of ctDNA for tissue DNA results.^a^

|  | | | **Stratified by time interval** | |  |
| --- | --- | --- | --- | --- | --- |
| **Parameters** | **Generator** | **All patients**  **(N=433)** | **≤2 months**  **(N=165)** | **>6 months**  **(N=199)** | **P-value** |
| *Sensitivity* | A / (A + B) | 54.0% | 55.2% | 51.2% | 0.65 |
| *Positive predictive value (true positive)* | A / (A + C) | 73.0% | 85.5% | 60.6% | 0.002 |
| *Specificity* | D / (C + D) | 80.3% | 87.0% | 75.7% | 0.09 |
| *Negative predictive value (true negative)* | D / (B + D) | 63.9% | 58.3% | 68.0% | 0.13 |

^a^**The table below was used to generate the numbers in Supplemental Table 4. Statistical differences in the specified parameters between two groups were determined by performing Fisher’s exact test.**

|  | Tissue | | |
| --- | --- | --- | --- |
|  |  | Positive | Negative |
| ctDNA | Positive | A | C |
|  | Negative | B | D |

**Supplemental Table 5:** Accuracy of tissue DNA for ctDNA results.^a^

|  | | | **Stratified by time interval** | |  |
| --- | --- | --- | --- | --- | --- |
| **Parameters** | **Generator** | **All patients**  **(N=433)** | **≤2 months**  **(N=165)** | **>6 months**  **(N=199)** | **P-value** |
| *Sensitivity* | A / (A + C) | 73.0% | 85.5% | 60.6% | 0.002 |
| *Positive predictive value* | A / (A + B) | 54.0% | 55.2% | 51.2% | 0.65 |
| *Specificity* | D / (B + D) | 63.9% | 58.3% | 68.0% | 0.13 |
| *Negative predictive value* | D / (C + D) | 80.3% | 87.0% | 75.7% | 0.09 |

^a^**The table below was used to generate the numbers in Supplemental Table 5. Statistical differences in the specified parameters between two groups were determined by performing Fisher’s exact test.**

|  | Tissue | | |
| --- | --- | --- | --- |
|  |  | Positive | Negative |
| ctDNA | Positive | A | C |
|  | Negative | B | D |

**Supplemental Figure 1.** Most common locus-specific alterations detected among 258 patients with *TP53* detected.^a^

**N=4,**

**40.0%**

| ***TP53* R248W**  Detection of *TP53* R248W in N=10 of 258 patients with *TP53* alterations.  **N=2,**  **22.2%** | **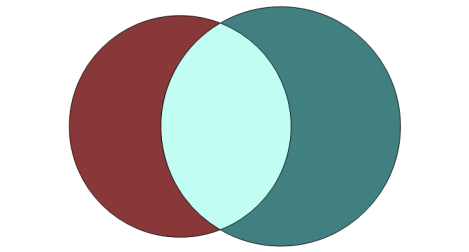**  **N=3,**  **33.3%**  **N=3,**  **30.0%**  **N=3,**  **30.0%** |
| --- | --- |
| ***TP53* R248Q**  Detection of *TP53* R248Q in N=9 of 258 patients with *TP53* alterations. | **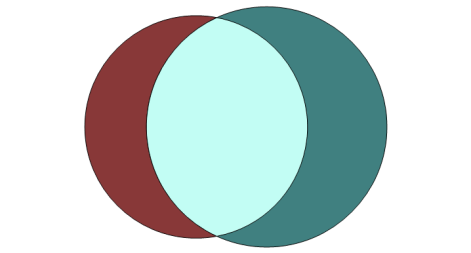**  **N=4,**  **44.4%** |
| ***TP53* R282W**  Detection of *TP53* R282W in N=9 of 258 patients with *TP53* alterations. | **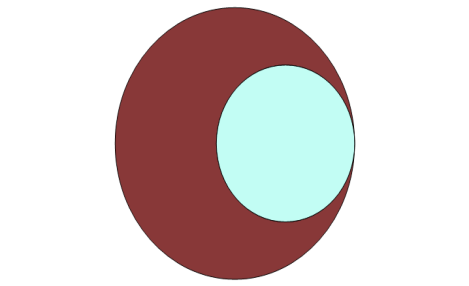**  **N=6,**  **66.7%**  **N=3,**  **33.3%** |
| ***TP53* G245S**  Detection of *TP53* G245S in N=8 of 258 patients with *TP53* alterations. | **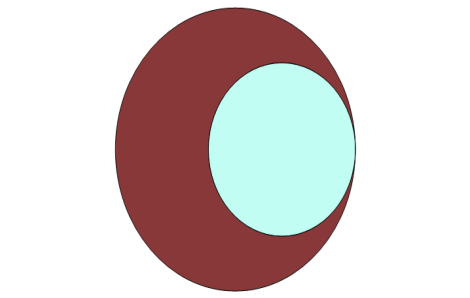**  **N=3,**  **37.5%**  **N=5,**  **62.5%** |
| ***TP53* R175H**  **N=2,**  **28.6%**  Detection of *TP53* R175H in N=7 of 258 patients with *TP53* alterations. | **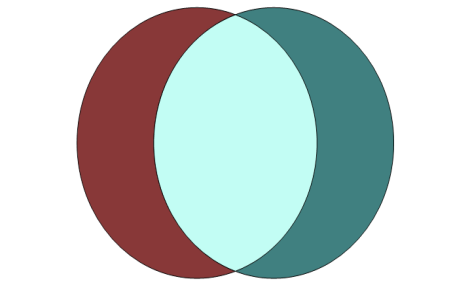**  **N=3,**  **42.9%**  **N=2,**  **28.6%** |

| *TP53* alterations in tissue only *TP53* alterations in ctDNA only *TP53* alterations in both tissue and ctDNA |
| --- |

^a^Venn diagrams represent the proportion of patients who had locus-specific *TP53* detected in only tissue, in both tissue and ctDNA, and only in ctDNA. Concordance was examined at the molecular level. If there was more than one time point of tissue or ctDNA NGS, the time points closest together for each patient was chosen.
